# Grape seed proanthocyanidin extract supplementation affects exhaustive exercise-induced fatigue in mice

**DOI:** 10.29219/fnr.v62.1421

**Published:** 2018-06-06

**Authors:** Liu Xianchu, Liu Ming, Liu Xiangbin, Zheng Lan

**Affiliations:** 1 Institute of Physical Education, Hunan University of Arts and Science, Hunan Province, Changde, China; 2 Key Laboratory of Physical Fitness and Exercise Rehabilitation of Hunan Province, Hunan Normal University, Changsha, China

**Keywords:** GSPE, exhaustive exercise, fatigue, anti-inflammatory, antioxidant, mitochondria

## Abstract

**Background:**

Grape seed proanthocyanidin extract (GSPE) has been extensively reported to possess a wide range of beneficial properties in multiple tissue damage. Previous studies have shown that exhaustive exercise-induced fatigue associates with oxidative stress injury, inflammatory response, and mitochondrial dysfunction.

**Objective:**

The aim of this study is to investigate the anti-fatigue effects of GSPE in mice and explore its possible underlying mechanism.

**Design:**

The mouse model of exhaustive exercise-induced fatigue was established by using the forced swimming test, and GSPE was orally treated for successive 28 days at 0, 1, 50 and 100 mg/kg/day of body weight, designated the control, GSPE-L, GSPE-M and GSPE-H groups, respectively.

**Results:**

The presented results showed that treatment of GSPE at a dose of 50 and 100 mg/kg/day of body weight significantly relieved exhaustive exercise-induced fatigue, indicated by increasing the forced swimming time. In addition, treatment of GSPE significantly improved the creatine phosphokinase and lactic dehydrogenase, as well as lactic acid level in exhaustive swimming. For underlying mechanisms, treatment of GSPE had anti-fatigue effects by promoting antioxidant ability and resisting oxidative effect, as represented by increased total antioxidative capability levels, enhanced superoxide dismutase and catalase activities, and ameliorated malondialdehyde levels. Furthermore, treatment of GSPE significantly inhibited the activity of tumor necrosis factor-α and interleukin-1β, which suggested that its protective effects on exhaustive exercise-induced fatigue may be attributed to inhibition of inflammatory response. Last but not the least, treatment of GSPE significantly improved succinate dehydrogenase and Na+-K+-ATPase levels to enhance mitochondrial function during exhaustive swimming-induced fatigue.

**Conclusions:**

These results proved that treatment of GSPE possessed the beneficial properties of anti-inflammatory, antioxidant, and mitochondrial protection to improve exhaustive exercise, which suggested that GSPE could be used as an effective functional food to delay fatigue.

Exercise involves human physiological changes (1, 2). Regular exercise has been widely demonstrated to have a function of lowering the risk of human chronic diseases, such as metabolic syndrome, cardiovascular disease, obesity, hypertension, type 2 diabetes, and even some types of cancers (3–5). However, exhaustive exercise is harmful, as it can cause muscle damage and also induce fatigue, which can be considered as extreme tiredness physically. Therefore, fatigue is a sub-health status and is closely associated with reducing the quality of life, which can result in fatigue syndrome and even lead to physical and mental unfitness (6, 7). Nowadays, fatigue as a complicated physiological phenomenon has become a serious phenomenon because of the huge pressures of life and work in the rapid development of society (8). So it is necessary and significant to seek effective measures to prevent and ease fatigue.

There are multiple reasons on the etiology of fatigue, such as exhaustion theory, homeostasis disturbance theory, and catastrophe theory (9). Among these, oxidative stress injury, inflammatory response, and mitochondrial dysfunction are widely known to play a critical role in the development of fatigue (10, 11). Nowadays, the development of natural products with the ability of reducing muscle injury and possessing anti-fatigue effects is becoming a major research focus (12). Previous studies have showed that *Dendrobium officinale* extract delays fatigue effects through enhancing glutathione peroxidase (GSH-Px) level and inhibiting malondialdehyde (MDA) content in serum (13). In the single exhaustive swimming test, dietary tea polyphenols can effectively protect against fatigue by inhibiting serum levels of tumor necrosis factor-α (TNF-α), interleukin-1β (IL-1β), and IL-6, improving IL 10/TNF-α ratio in serum and reducing IL-1β mRNA expression in liver (14). In addition, dietary nucleotides possess the function of improving mitochondrial biogenesis, including succinate dehydrogenase (SDH), Na+-K+-ATPase, and Ca2+-Mg2+-ATPase, in skeletal muscles, to exert anti-fatigue effects (15). Therefore, nutritional intervention on antioxidant and anti-inflammatory, as well as mitochondrial protection is a valuable approach to fight against fatigue.

Grape is one of the most popular fruits in the world, and proanthocyanidin is the main component of grape seed with various pharmacological effects (16, 17). More and more researches have showed that grape seed proanthocyanidin extract (GSPE) has been used for the treatment of numerous diseases because of its therapeutic activities, such as anti-inflammatory and antioxidant properties (18). Previous studies have showed that natural proanthocyanidin-rich extracts from grape seed exhibits an antioxidant effect in the gastrointestinal tract, as represented by reduced intestinal ROS in fasted animals (19, 20). On the subchronic immune injury, intervention of GSPE could significantly decrease the expression of TNF-α, IL-1β, IL-6, and IFN-γ to restrain inflammatory reaction (21). Moreover, GSPE can significantly improve indoxyl sulfate-induced HUVEC (human umbilical vein endothelial cells) injury by ameliorating mitochondrial dysfunction (22). Although previous studies have demonstrated that grape seed extract is involved in regulating oxidative stress on the treadmill running performance (23), there is no prior report on the protective effects of proanthocyanidins from grape seed on exhaustive exercise. Furthermore, the anti-fatigue mechanism of GSPE remains to be observed deeply. The purpose of this study was to study the relationship between GSPE and exhaustive exercise, and further clarify the possible protective effects of GSPE on fatigue.

## Materials and methods

### Animals

Male ICR mice (about 8 weeks old, 25 ± 2 g) were obtained from the Animal Center of Hunan Normal University (Changsha, China). All mice were grown at a standard laboratory condition (23 ± 2°C, 50–60% humidity, 12-h light/12-h dark cycle). The mice were randomly divided and housed in groups of four animals per cage. They were provided with free access to standard diet and distilled water. In this study, all animal experiments were inspected according to the Animal Care Committee of Hunan Normal University.

GSPE was purchased from Tianjin Jianfeng Natural Product R&D Co., LTD (purity: ≥95%, Tianjin, China). Thirty-two male ICR mice were randomly assigned to four experimental groups (8 mice per group) for GSPE treatment: (1) control group, (2) low-dose group (GSPE-L), (3) medium-dose group (GSPE-M), and (4) high-dose group (GSPE-H). Three GSPE intervention groups were treated with GSPE at a dose of 1, 50 and 100 mg/kg/day of body weight, respectively. The control group mice were administrated at the same dosage volume of physiological saline equivalent to individual body weight. GSPE supplementation was given by oral gavages once a day for 28 successive days.

### Forced swimming test

The mice were challenged by the forced swimming test to establish exhaustive exercise-induced fatigue. One hour after the final dosing, all mice underwent weight-loaded swimming test with a lead (approximately 5% of each mouse’s body weight), which was attached to their tails root. All mice were trained individually in the same condition (25 ± 1°C, 30 cm depth) (25). Before being subjected to the forced swimming test, all mice were also drilled to adapt to swimming without any loads for 3 days (20 min/day). The time to exhaustion (TTE), which was defined by failure to rise to the above water within 10 s and a lack of apparent coordinated movements, was recorded immediately. After the exhaustive swimming, the mice were killed by an intraperitoneal injection of anesthetic. Then blood and skeletal muscle tissues were collected and removed for further experiments.

### Biochemical assay

Blood samples were obtained and centrifuged at 1,200 × g and 4°C for 15 min to separate serum after the swimming exercise. The serum biochemical variables related to fatigue (Catalog number: A019 for lactic acid [LA], A020 for lactic dehydrogenase [LDH], A032 for creatine phosphokinase [CK], Nanjing Jiancheng Biotechnology Institute, Nanjing, China) were measured according to the manufacturer’s protocol.

### Determination of oxidative stress

After the swimming exercise, skeletal muscle tissues were immediately acquired and stored at −80°C. In our research, skeletal muscle tissues were carefully grounded and centrifuged. Then the serum and supernatant in skeletal muscle were used for oxidative stress assay. Furthermore, the level of antioxidant enzymes and oxidative indicators (Catalog number: A001 for superoxide dismutase [SOD], A003 for MDA, A007 for catalase [CAT], A015 for total antioxidative capability [T-AOC], Nanjing Jiancheng Biotechnology Institute, Nanjing, China) were determined by spectrophotometer according to the manufacturer’s protocol.

### Examination of inflammatory response

The level of inflammatory cytokines (Catalog number: EK0527 for TNF-α, EK0411 EK0394 for IL-1β, BOSTER Biological Technology, Wuhan, China) were examined to assess inflammatory responses in the serum and supernatant of skeletal muscle by ELISA. The absorbance value was measured at 450 nm to analyze inflammatory parameters according to the manufacturer’s instructions.

### Measurements of energy metabolism

The energy metabolism parameters SDH and Na+K+-ATPase (Catalog number: A022 for SDH, A016 for Na+K+-ATPase, Nanjing Jiancheng Biotechnology Institute, Nanjing, China) in skeletal muscles were determined to assess mitochondrial function by detection kits according to the manufacturer’s protocol.

### Statistics

All results were displayed as mean ± standard deviation (SD). The data was statistically analyzed using SPSS 16.0 software. Statistical significance between groups was demonstrated with one-way analysis of variance (ANOVA). A *p* value of less than 0.05 was considered statistically significant.

## Results

### Effects of GSPE on body weight of mice

The body weight of mice in our experiment is presented in Table 1. The general condition of all mice was normal and the survival rate was 100% during the whole experimental process. There were no remarkable differences among the groups on the initial body weight. After GSPE supplementation for 28 successive days, the terminal weight in mice showed that the control, GSPE-L, GSPE-M, and GSPE-H groups were 38.82 ± 1.70, 37.66 ± 1.81, 36.91 ± 2.11, and 38.30 ± 1.80 g, respectively, which displayed that there were no remarkable differences in final body weight in the control and GSPE groups.

**Table 1:** Effects of grape seed proanthocyanidin extract on the body weight and time to exhaustion in mice

Parameters	Control		GSPE-L		GSPE-M		GSPE-H	
	Mean	SD	Mean	SD	Mean	SD	Mean	SD
Initial body weight (g)	25.56	0.70	25.14	0.90	25.25	1.36	25.29	1.28
Final weight (g)	38.82	1.70	37.66	1.81	36.91	2.11	38.30	1.80
Exhaustive time (min)	8.60	1.46	9.79	1.90	13.03**	3.50	16.63**	2.98

Note: Data were displayed as means ± SD.

** *p* < 0.01, versus control group. GPSE-L, dietary grape seed proanthocyanidin extract low-dose group; GPSE-M, dietary grape seed proanthocyanidin extract medium-dose group; GPSE-H, dietary grape seed proanthocyanidin extract high-dose group; SD, standard deviation.

### Effects of GSPE in the weight-loaded swimming test

The effects of GSPE on the weight-loaded swimming time are presented in Table 1. Our results displayed that the TTE of the control, GSPE-L, GSPE-M, and GSPE-H groups was 8.60 ± 1.46, 10.29 ± 2.24, 13.03 ± 3.50, and 16.63 ± 2.98, respectively. When compared to the control group, the TTE in GSPE groups increased by 19.65, 51.51, and 93.37%, respectively, and the difference was markedly significant in the GSPE-M and GSPE-H groups.

### Effects of GSPE on LA, CK, and LDH in serum

The effects of GSPE on the blood biochemical parameters are presented in Table 2. In comparison with the control group, the LA levels in serum were significantly increased in GSPE-M and GSPE-H groups after exhaustive swimming. Moreover, the CK and LDH activity in serum was also measured to evaluate the protective effects of GSPE on the tissue damage induced by exhaustive swimming. In this study, the serum CK and LDH activities in GSPE-M and GSPE-H groups were significantly lower than those in the control group.

**Table 2:** Effects of grape seed proanthocyanidin extract on biochemical indexes in serum of mice

Parameters	Control		GSPE-L		GSPE-M		GSPE-H	
	Mean	SD	Mean	SD	Mean	SD	Mean	SD
LA (mmol/L)	12.44	2.75	10.80	1.51	9.51*	1.19	8.74**	1.35
CK(U/mL)	1.42	0.15	1.29	0.14	1.06**	0.23	0.85**	0.17
LDH(U/mL)	5.33	0.19	5.09	0.26	4.74**	0.47	4.24**	0.40

Note: Data were displayed as means ± SD.

* *p* < 0.05,

** *p* < 0.01, versus control group. GPSE-L, dietary grape seed proanthocyanidin extract low-dose group; GPSE-M, dietary grape seed proanthocyanidin extract medium-dose group; GPSE-H, dietary grape seed proanthocyanidin extract high-dose group; SD, standard deviation.

### Effects of GSPE on parameters of oxidative stress in serum and skeletal muscles of mice

The effects of GSPE on oxidative stress in both serum and skeletal muscles of mice are presented in Table 3. In the comparison with the control group, the activities of SOD and CAT were significantly increased in GSPE-M and GSPE-H groups. In addition, T-AOC levels were significantly enhanced, while MDA levels were significantly improved, in GSPE-M and GSPE-H groups compared with the control group.

**Table 3:** Effects of grape seed proanthocyanidin extract on oxidative stress parameters in serum and skeletal muscles of mice

	Parameters	Control		GSPE-L		GSPE-M		GSPE-H	
		Mean	SD	Mean	SD	Mean	SD	Mean	SD
Serum	T-AOC (U/mL)	117.75	12.14	121.62	9.66	139.76**	10.68	139.49**	9.07
	SOD (U/mL)	71.82	12.02	76.86	6.17	84.49**	7.67	88.11**	6.32
	CAT (U/mL)	24.63	3.34	27.71	2.58	27.97*	2.83	28.97**	2.30
	MDA (nmoL/mL)	8.36	0.68	7.93	0.82	7.19*	1.24	6.77**	0.95
Skeletal muscle	T-AOC (U/mg pro)	4.08	0.50	4.39	0.52	5.01**	0.54	5.04**	0.64
	SOD (U/mg pro)	7.19	0.72	7.95	0.98	8.12*	0.65	9.83**	1.00
	CAT (U/mg pro)	3.50	0.78	4.08	0.60	4.71**	0.45	4.74**	0.49
	MDA (nmoL/mg pro)	4.74	0.44	4.39	0.36	4.29*	0.39	3.75**	0.76

Note: Data are displayed as means ± SD.

* *p* < 0.05,

** *p* < 0.01, versus control group. GPSE-L, dietary grape seed proanthocyanidin extract low-dose group; GPSE-M, dietary grape seed proanthocyanidin extract medium-dose group; GPSE-H, dietary grape seed proanthocyanidin extract high-dose group; SD, standard deviation.

### Effects of GSPE on activities of inflammatory response in serum and skeletal muscles of mice

Inflammatory cytokines, including TNF-α and IL-1β, in both serum and skeletal muscles of mice were measured to evaluate the effects of GSPE on inflammatory response. As shown in Table 4, the TNF-α and IL-1β levels of mice were improved in GSPE-M and GSPE-H groups with remarkable differences in comparison with the control group after exhaustive exercise.

**Table 4:** Effects of grape seed proanthocyanidin extract on inflammatory response in serum and skeletal muscles of mice

	Parameters	Control		GSPE-L		GSPE-M		GSPE-H	
		Mean	SD	Mean	SD	Mean	SD	Mean	SD
Serum	TNF-α (pg/mL)	153.88	7.98	146.71	10.24	143.89*	9.60	140.41**	7.91
	IL-1β (pg/mL)	75.70	3.42	72.48	4.15	70.06*	4.46	69.57**	3.19
Skeletal muscle	TNF-α (pg/mg pro)	73.72	4.02	70.98	3.90	67.12**	3.06	65.12**	3.92
	IL-1β (pg/mg pro)	27.39	1.62	25.94	1.29	24.85**	1.28	24.65**	2.02

Note: Data are displayed as means ± SD.

* *p* < 0.05,

** *p* < 0.01, versus control group. GPSE-L, dietary grape seed proanthocyanidin extract low-dose group; GPSE-M, dietary grape seed proanthocyanidin extract medium-dose group; GPSE-H, dietary grape seed proanthocyanidin extract high-dose group; SD, standard deviation.

### Effect of GSPE on activities of mitochondrial function in skeletal muscles

The SDH and Na+-K+-ATPase activity were measured to evaluate the effects of GSPE on mitochondrial function in skeletal muscles. As shown in Table 5, when compared with the control group, the SDH and Na+-K+-ATPase activities in skeletal muscle were markedly increased in the GSPE-M and GSPE-H groups.

**Table 5:** Effects of grape seed proanthocyanidin extract on mitochondrial function in skeletal muscles of mice

Parameters	Control		GSPE-L		GSPE-M		GSPE-H	
	Mean	SD	Mean	SD	Mean	SD	Mean	SD
SDH (U/mg pro)	2.52	0.28	2.70	0.34	2.91*	0.38	2.91*	0.27
Na+K+-ATPase (U/mg pro)	0.83	0.11	0.94	0.10	1.03**	0.12	1.06**	0.13

Note: Data are displayed as means ± SD.

* *p* < 0.05,

** *p* < 0.01, versus control group. GPSE-L, dietary grape seed proanthocyanidin extract low-dose group; GPSE-M, dietary grape seed proanthocyanidin extract medium-dose group; GPSE-H, dietary grape seed proanthocyanidin extract high-dose group; SD, standard deviation.

## Discussion

Grape is a popular fruit throughout the world. Nowadays, proanthocyanidin, as major flavonoids in grape seed, is used as a nutritional supplementation worldwide for its medicinal and health effects (26, 27). It has been suggested that grape seed extract supplementation improves oxidative stress by preventing MDA levels and increasing antioxidant enzyme activities to prevent exercise-induced oxidative damage (23, 28). In this study, proanthocyanidin from grape seed was hypothesized to bring beneficial health effects against exhaustive exercise-induced fatigue because of its notable effects of inhibiting oxidative stress and inflammatory response, as well as potentiating mitochondrial function. The mouse model of exhaustive exercise was established to assess the anti-fatigue effects of GSPE by forced swimming test and to illuminate its potential mechanism. In general, the ability of delaying fatigue is positively correlated with the TTE in forced swimming test (29). In this study, our results showed that treatment with GSPE improved the fatigue. GSPE supplementation prolonged the TTE, particularly at 19.65, 51.51, and 93.37% in GSPE-treated groups, which may indicate that GSPE is capable of delaying fatigue. Furthermore, our results showed that GSPE supplementation did not significantly cause body weight changes in either the control group or the GSPE group, which was consistent with the previous reports (30). In summary, there was no abnormality and no death occurred in this study, which indicates that GSPE does not have any toxicity and is an anti-fatigue natural agent.

LA is formed as a metabolic by-product in skeletal muscles and released into the bloodstream. However, excess LA is harmful for skeletal muscles during intense exercise, which may result in fatigue (31). Therefore, LA is identified as a biochemical index relevant to fatigue in blood. CK and LDH are muscle injury indicators clinically (32, 33). There is a prominent positive correlation between the activity of CK and LDH and the process of fatigue. To further evaluate the protective effect of GSPE on exhaustive swimming-induced fatigue, the fatigue indicators, including LA, CK, and LDH, were tested in our study. GSPE could lower the contents of LA and decrease the activity of LDH and CK in serum, which indicates that GSPE is capable of protecting skeletal muscles from damage and delaying fatigue in exhaustive exercise.

Exhaustive exercise leads to oxidative stress, which can destabilize the balance between oxidation and antioxidation systems and play an important role in fatigue-related disorders (34, 35). It is also believed that antioxidant enzymes play an important role in defensing against oxidative stress. SOD and CAT belong to antioxidant enzymes, while MDA is a part of oxidative indicators and aggravates oxidative damage. The anomaly between oxidation and antioxidation is associated with the development of fatigue progress. Previous literature has showed that okra seeds promote antioxidant ability through increasing SOD and GSH-PX levels and lowering MDA level to possess anti-fatigue effects (9). In addition, GSPE significantly improved oxidative stress by enhancing SOD and CAT levels and reducing MDA contents in spontaneously hypertensive rats (36). In this study, our results showed that GSPE increased the activity of SOD and CAT and inhibited MDA contents in exhaustive exercise, indicating that anti-fatigue mechanism of GSPE was at least partly associated with its antioxidant effects.

Exhaustive exercise has been reported to cause inflammatory response which can contribute to fatigue. TNF-α, IL-6, and IL-1β are often used as an index to evaluate the inflammatory response. Glycine-leucine and leucine-glycine dipeptides, as primary peptides of fermented porcine placenta, significantly decreased IL-6 and IL-1β serum levels to display an anti-fatigue effect (37). Furthermore, oxidative stress is recognized as a primary mediator of inflammatory response. Hence, anti-inflammatory effect as a part of nutritional intervention is an indispensable part of prevention and improvement of fatigue. Thus far, relatively few studies have demonstrated whether GSPE can possess anti-fatigue function through affecting inflammatory cytokine activities during exhaustive exercise, although its anti-inflammatory function has been reported on multiple diseases in animal experiments and clinical studies. In this study, the elevated inflammatory cytokine activity induced by exhaustive exercise in blood, which was represented by increasing the level of TNF-α and IL-1β, was noticeably alleviated by GSPE pretreatment. Moreover, excess inflammatory cytokine in skeletal muscle can induce tissue injury and further accelerate fatigue. Our results also showed that GSPE decreased the activity of TNF-α and IL-1β in the skeletal muscle. These data well suggested that anti-fatigue effects of GSPE might be related to its anti-inflammatory function.

Mitochondrion is the place of adenosine triphosphate (ATP) generation. The human body needs more ATP to maintain muscle contraction and movement during exercise. However, exhaustive exercise can lead to damaged mitochondria that further cause skeletal muscles damage and fatigue (38). SDH plays an important role in ATP synthesis, while Na+-K+-ATPase can reflect ATP hydrolysis during energy supply to maintain normal physiologic function (39, 40). Therefore, SDH and Na+-K+-ATPase are the primary factors responsible for energy metabolism in mitochondria, the disorder activity of which contributes to exhaustive swimming-induced fatigue. Previous findings indicate that GSPE promotes mitochondrial oxygen consumption and the enzyme activity of citric acid cycle and electron transport chain (ETC) to affect the mitochondrial function and energy metabolism (41). To elucidate the anti-fatigue effect of GSPE after exhaustive swimming, the activities of SDH and Na+-K+-ATPase in skeletal muscles were measured to assess mitochondrial function. Our results showed that GSPE could improve mitochondrial function for energy supplementation, as represented by enhancing levels of SDH and Na+-K+-ATPase, to delay fatigue during exhaustive swimming.

## Conclusion

To our knowledge, this research is the first to investigate the effect of GSPE supplementation on exhaustive swimming-induced fatigue in mice. Treatment with GSPE improved the fatigue by prolonging the TTE in the forced swimming test. Furthermore, several biochemical markers for fatigue, including LA, LDH, and CK, were significantly alleviated by the administration of GSPE. The anti-fatigue property may be associated with the amelioration of inflammatory response, suppression of oxidative stress, and improvement of mitochondrial function. According to the data, these findings indicate that GSPE is a natural and safe ingredient and dietary agent in excessive exercise-induced fatigue.
